# Impact of pre-bypass ultrafiltration on prime values and clinical outcomes in neonatal and infant cardiopulmonary bypass

**DOI:** 10.1051/ject/2023039

**Published:** 2023-12-15

**Authors:** Katherine Kohlsaat, Kimberlee Gauvreau, Francis Fynn-Thompson, Sharon Boyle, Kevin Connor, William Regan, Gregory Matte, Meena Nathan

**Affiliations:** 1 Department of Cardiac Surgery, Boston Children’s Hospital Boston MA USA; 2 Department of Cardiology, Boston Children’s Hospital Boston MA USA; 3 Department of Biostatistics, Harvard School of Public Health Boston MA USA; 4 Department of Surgery, Harvard Medical School Boston MA USA

**Keywords:** Pre-bypass ultrafiltration, Congenital, PBUF, Electrolytes, Outcomes, Bypass prime, Blood prime, Prime

## Abstract

*Background*: A standard blood prime for cardiopulmonary bypass (CPB) in congenital cardiac surgery may possess non-physiologic values for electrolytes, glucose, and lactate. Pre-bypass Ultrafiltration (PBUF) can make these values more physiologic and standardized prior to bypass initiation. We aimed to determine if using PBUF on blood primes including packed red blood cells and thawed plasma would make prime values more predictable and physiologic. Additionally, we aimed to evaluate whether the addition of PBUF had an impact on outcome measures. *Methods*: Retrospective review of consecutive patients ≤ 1 year of age undergoing an index cardiac operation on CPB between 8/2017 and 9/2021. As PBUF was performed at the perfusionists’ discretion, a natural grouping of patients that received PBUF vs. those that did not occur. Differences in electrolytes, glucose, and lactate were compared at specific time points using Fisher’s exact test for categorical variables and the Wilcoxon rank sum test for continuous variables. Clinical outcomes were also assessed. *Results*: In both cohorts, the median age at surgery was 3 months and 47% of patients were female; 308/704 (44%) of the PBUF group and 163/414 (39%) of the standard prime group had at least one preoperative risk factor. The proportion of PBUF circuits which demonstrated more physiologic values for glucose (318 [45%]), sodium (434, [62%]), potassium (688 [98%]), lactate (612 [87%]) and osmolality (595 [92%]) was significantly higher when compared to standard prime circuit levels for glucose (8 [2%]), sodium (13 [3%], potassium (150 [36%]), lactate (56 [13%]) and osmolality (23 [6%]) prior to CPB initiation. There were no differences in clinical outcomes or rates of major adverse events between the two cohorts. *Conclusions*: PBUF creates standardized and more physiologic values for electrolytes, glucose, and lactate before the initiation of bypass without significant impacts on in-hospital outcomes.

## Introduction

Blood priming a cardiopulmonary bypass (CPB) circuit may be necessary for cardiac surgery, especially in the neonatal and infant population. Banked blood and its component products are known to have non-physiologic values for many electrolytes, glucose, and lactate due to preservative and anticoagulant additives as well as changes that occur during refrigerated storage [1]. This can lead to undesirable hemodynamic effects once transfused [2–5]. Cell saver washing of packed red blood cell (PRBC) units has been shown to effectively control mediators of hemodynamic change seen with stored blood [3–6]. However, increased cell wall fragility and hemolysis after processing and during bypass may be seen [6, 7]. This can lead to excess free iron in the blood which may then bring about oxidative stress, an increased risk of infection, and other negative outcomes [8–13]. It is important to note that cell-saver washing of PRBCs does not address nonphysiologic values in thawed plasma when that blood product is added to a blood prime. Pre-bypass ultrafiltration (PBUF) may be used to correct known issues with banked blood (PRBCs with or without plasma) for patients requiring a blood prime of the CPB circuit [14–19]. When a blood prime is indicated, this technique is used prior to CPB initiation in order to create more physiologic electrolytes, glucose, and lactate values [15]. The PBUF process importantly creates a way to have more standardized prime values when using banked blood.

The use of PBUF has been shown to attenuate cardiac impairment seen during the early reperfusion periods and to reduce pulmonary dysfunction in neonates [13]. Delaney et al. reported on the impact PBUF had on potassium concentration [15]. To reduce the risk of transfusion-related hyperkalemic cardiac arrests in small children, they measured analytes at four designated time points. They found that the age of the red blood cell (RBC) unit was linearly associated with increased potassium concentration, but that the mean potassium concentration decreased from 10.9 to 6.0 mEq/L (*p* = 0.001) utilizing their technique of PBUF processing [15].

The use of PBUF can also slow down the activation of the coagulation pathway and attenuate the inflammatory response [15, 16, 20]. Nagashima et al. saw significantly reduced concentrations of bradykinin and high molecular weight kininogen following the use of PBUF in neonates undergoing an arterial switch operation, highlighting a possible advantage of PBUF in patients who require a blood prime for CPB [16].

The American Society of Extracorporeal Technology (AmSECT) is the primary national organization for perfusionists. AmSECT published *Standards and Guidelines for Pediatric and Congenital Perfusion Practice* in 2019 [21]. Standard 13.2 states that the perfusionist shall correct any physiologic abnormalities in blood-primed circuits. Additionally, guideline 13.1 recommends prebypass ultrafiltration as one method to achieve the standard. The process of blood priming the CPB circuit is not standardized across institutions and there are several methods used to correct for abnormal prime values [18]. We hypothesized that our method of PBUF would normalize and standardize blood primes with more consistent values prior to initiation of CPB without adversely affecting clinical outcomes.

### Objectives

We aimed to determine the effects of the addition of PBUF to CPB circuits requiring a blood prime for neonates and infants. Our institution had a long-standing standardized method of blood priming. Some perfusionists elected to PBUF all of their circuits after a standard blood prime, while others did not out of concern for unknown effects. A multidisciplinary meeting of perfusionists, surgeons, anesthesiologists, and nurses determined that the primary concern centered on the use of 0.45% saline in the process, which could potentially lyse RBCs and increase plasma-free hemoglobin. Lowering the prime sodium level closer to its physiologic range also had the potential to increase edema after CPB. Additionally, there were concerns that changing practice may lead to an increase in adverse events and have a negative impact on patient outcomes. Our institutional practices allowed for a natural trial in that some perfusionists utilized PBUF while others maintained the standard priming technique due to the above concerns.

## Materials and methods

This retrospective cohort study included all consecutive infants at our institution under one year of age undergoing cardiac surgery with CPB between August 2017 and September 2021. The index procedure of each hospitalization was included. The study was exempted by the Institutional Review Board (#IRB-P00038496) with a waiver of consent. The primary aim of the study was to determine how effective PBUF was at normalizing blood prime electrolyte, glucose, and lactate values prior to the initiation of bypass. The secondary aim was to determine if PBUF was associated with any adverse intraoperative or postoperative outcomes.

### Outcomes, primary predictors, and covariates

The primary outcome was the difference in sodium, potassium, glucose, and lactate levels between the standard prime (SP) cohort and the PBUF cohort during the intraoperative period. The time points of interest included (a) first prime values (standard prime in both groups), (b) final prime values (different only in the PBUF cohort), (c) first values measured after bypass initiation, and (d) last values measured prior to CPB cessation. We also looked at differences in osmolality between the two cohorts, particularly at time point (b). Secondary outcomes included differences in clinical outcomes between the PBUF and SP cohorts. The clinical outcomes measured included postoperative ventilation duration, cardiac intensive care unit (CICU) length of stay (LOS), postoperative hospital LOS, and major adverse events (unplanned reoperation, re-exploration for bleeding, mediastinitis, central nervous system (CNS) complications, extracorporeal membrane oxygenation (ECMO), pacemaker, and mortality). We also examined phenylephrine use on bypass, total ultrafiltration volume, and inotrope requirements.

The primary predictor was the use of PBUF. Other preoperative covariates included age; sex; case complexity as measured by the Society of Thoracic Surgeons-European Association for Cardio-Thoracic Surgery (STAT) mortality categories; prematurity; noncardiac anomalies/chromosomal abnormalities/syndromes; and preoperative risk factors as defined in the STS Congenital Heart Surgery Database data collection form (mechanical ventilation, ECMO, renal dysfunction, cardiopulmonary resuscitation, stroke, sepsis, seizures, hepatic dysfunction, necrotizing enterocolitis). We also collected information on whether the patient had prior cardiac surgery, whether the index operation was a palliative surgery versus a complete repair resulting in biventricular circulation, as well as the urgency status of the surgery categorized as elective, urgent, emergent, or salvage.

Intraoperative covariates included total CPB time, aortic cross-clamp time, circulatory arrest time, and adequacy of repair as measured by intraoperative and postoperative Technical Performance Score (TPS) [22].

### Cardiopulmonary bypass circuit priming

All CPB cases utilized a circuit consisting of a CAPIOX FX-05 oxygenator (Terumo Cardiovascular, Inc., Ann Arbor, MI), a custom tubing pack (LivaNova PLC, London, UK) and an HPH400 hemoconcentrator (Medivators Inc. Minneapolis, MN). The circuit prime volume, inclusive of the 70 mL active ultrafiltration circuit and 75 mL in the reservoir was 285 mL for circuits with a 3/16″ arterial boot and 300 mL for circuits with a 1/4″ arterial boot. All patients received 30 mg/kg of methylprednisolone at the onset of bypass per our institutional standard as well as antibiotic coverage. A natural grouping of patients that received PBUF versus those that did not occur as PBUF was performed at the perfusionist’s discretion. The SP control group included all patients who received a standard blood prime. The PBUF group received a standard blood prime followed by the PBUF procedure, which was performed before the initiation of CPB.

A standard blood prime consisted of the perfusionist flushing the circuit with carbon dioxide before proceeding to prime the circuit with Plasma-Lyte A 7.4 (Baxter Healthcare, Deerfield, IL) heparinized to 3 IU/mL. Then, the majority portion of the crystalloid in the circuit was displaced with blood-bank-reconstituted whole blood (1 unit of supernatant-removed-RBCs mixed with one unit of plasma), also heparinized to 3 IU/mL. Sodium bicarbonate (6 [±1] mEq) and calcium gluconate (600 [±100] mg) were then added to normalize pH and ionized calcium levels, respectively. Blood gas analysis was performed on the prime blood before patient use.

The PBUF prime group received a standard blood prime followed by the PBUF procedure. The PBUF process included the addition of 200 mL of heparinized Plasma-Lyte A and 80 mL of 0.45% saline to the cardiotomy venous reservoir. This excess volume was then filtered off, bringing the reservoir level back to 75 mL. Additional sodium bicarbonate and calcium gluconate were added to normalize values and blood gas analysis confirmed the results before patient use. Please see the Limitations section for the post-study updated protocol that includes PBUF of the entire unit of reconstituted blood with sequestering of volume not needed in the prime for transfusion later during the case.

### Statistical analysis

Categorical variables are summarized with frequencies and percentages, and continuous variables with ranges or interquartile ranges as noted. Patient characteristics, clinical variables, and outcomes were compared for patients receiving and not receiving PBUF using Fisher’s exact test or the Wilcoxon rank sum test. Summaries were also stratified by case complexity. Analyses were conducted in Stata version 16 (StataCorp, College Station, TX).

## Results

### Baseline patient characteristics

Between August 2017 and September 2021, 704 patients under 1 year of age underwent open-heart surgery for a congenital heart defect where PBUF was utilized. In contrast, 417 patients under 1 year of age during this period did not undergo PBUF. Baseline patient characteristics are described in Table 1. While there are no statistically significant differences in these baseline patient characteristics, it is notable that the median age at surgery was 3 months and 47% of patients were female in both cohorts. Preoperative risk factors were present in 308 (44%) of the PBUF cohort and 163 (39%) of the SP cohort (Table 1). At the recorded index surgery, 149 (21%) of the PBUF cohort and 97 (23%) of the SP cohort had previously undergone a prior cardiac operation with or without CPB (Table 1). The median cardiopulmonary bypass times (CPBT) were similar with a CPBT of 141 [IQR 103–197] min in the SP group and 145 [IQR 104–198] min in the PBUF group. Cross-clamp time (CCT) was also similar between groups at 91 [IQR 54–128] min in the SP group and 93 [IQR 60–125] min in the PBUF group.

**Table 1:** Patient characteristics/demographics.

	Standard prime (*n* = 417)	PBUF (*n* = 704)	*P*-value
Age at surgery (months)	3 [25d, 6]	3 [8d, 6]	0.086
Age at surgery ≤30 days	109 (26%)	216 (31%)	0.12
Sex female	195 (47%)	328 (47%)	1.0
Premature^1^	98 (24%)	139 (20%)	0.15
Palliative procedure	82 (20%)	156 (22%)	0.36
≥1 prior surgery with CPB	67 (16%)	98 (14%)	0.34
≥1 prior surgery without CPB	30 (7%)	51 (7%)	1.0
Noncardiac anomaly	115 (28%)	206 (29%)	0.58
Chromosomal abnormality	127 (30%)	221 (31%)	0.79
Syndrome	138 (33%)	221 (31%)	0.60
Preoperative risk factor^2^	163 (39%)	308 (44%)	0.13
STAT mortality category			
1	50 (12%)	100 (14%)	0.045
2	76 (18%)	108 (15%)	
3	85 (20%)	170 (24%)	
4	172 (41%)	249 (35%)	
5	28 (7%)	72 (10%)	
Not assigned	6 (1%)	5 (1%)	
Urgency status (*n* = 413, 702)			
Elective	212 (51%)	319 (45%)	0.075
Urgent	195 (47%)	377 (54%)	
Emergency	6 (1%)	5 (1%)	
Salvage	0 (0%)	1 (<1%)	
Cardiopulmonary bypass time (min) (*n* = 417, 703)	141 [103, 197]	145 [104, 198]	0.50
Cross-clamp time (min) (*n* = 410, 688)	91 [54, 128]	93 [60, 125]	0.66
Any circulatory arrest time (*n* = 410, 692)	75 (18%)	128 (19%)	1.0
Operating room time (min)	407 [338, 492]	416 [341, 499]	0.69
Intraoperative TPS^3^			
1	279 (67%)	491 (70%)	0.55
2	122 (29%)	190 (27%)	
3	5 (1%)	11 (2%)	
4 not assigned	11 (3%)	12 (2%)	
TPS at discharge^4^			
1	204 (49%)	367 (52%)	0.44
2	118 (28%)	204 (29%)	
3	79 (19%)	113 (16%)	
4 Not assigned	16 (4%)	20 (3%)	

Values shown are number (percent) or median [25th, 75th percentiles].

^1^Less than 37 gestational weeks.

^2^Preoperative risk factors include: mechanical ventilation, ECMO, renal dysfunction, cardiopulmonary resuscitation, stroke, sepsis, seizures, hepatic dysfunction, necrotizing enterocolitis, arrhythmias, colostomy, gastrostomy tube, acidosis, hypothyroidism, bronchopulmonary dysplasia, hypo/hyper coagulable, pulmonary hypertension, adrenal insufficiency, tracheostomy, enterostomy, familial history of ischemic heart disease, endocarditis, asthma, implanted defibrillator, pacemaker, bronchiolitis, single lung, diabetes mellitus type 1.

^3^TPS is classified as Class 1: no residua; Class 2: minor residua; Class 3: major residua.

^4^TPS is classified as Class 1: no residua; Class 2: minor residua; Class 3: major residua or reintervention for residua prior to discharge.

CPB: cardiopulmonary bypass; Min: minutes; PBUF: pre-bypass ultrafiltration; STAT: Society of Thoracic Surgeons-European Association for Cardio-Thoracic Surgery; TPS: technical performance score.

### Primary outcome

**Figure 1:**
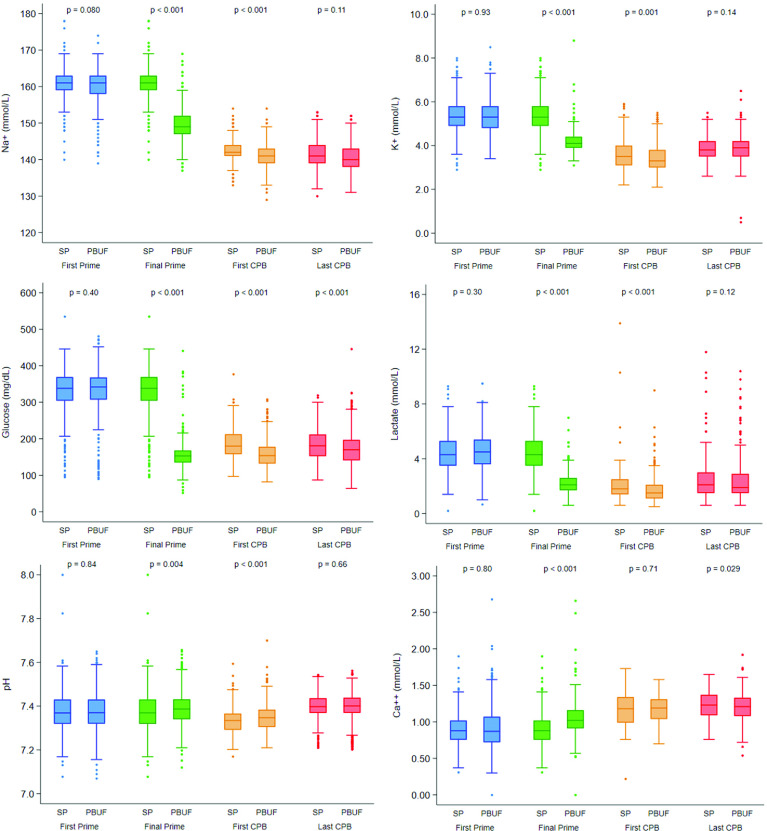
Electrolytes, glucose, and lactate measurements throughout the perioperative period. Distribution of electrolytes, glucose, and lactate measurements are displayed as box and whisker plots for both the standard prime and PBUF cohorts. The lower and upper borders of the box represent the 25th and 75th percentiles. The middle horizontal lines represent the median. The lower and upper whiskers represent the minimum and maximum values of non-outliers, while extra dots represent outliers. It is important to note that in the panel depicting lactate values, an outlier with a value > 20 mmol/L is not shown, as it would greatly shrink the box plot by expanding the scale. Furthermore, pH for prime values was measured at a temperature of 30 °C. pH on bypass was measured at 37 °C (alpha-stat) for all cases performed at >30 °C. pH on bypass was measured at the target temperature (pH-stat) for all cases ≤30 °C. For both cohorts, blue represents the first prime values, green represents the final prime values after PBUF (the same measurements are used for the blue and green groups for the standard prime cohort), orange represents the first CPB values after bypass initiation, and red represents the last CPB values prior to bypass cessation.

The SP and PBUF groups had similar prime values after each received a standard blood prime (Table 2), as expected. The PBUF cohort had statistically significant shifts towards physiologic values for sodium, glucose, potassium, lactate, and osmolality after that procedure was added (Tables 3a and 3b). While osmolality in the two cohorts was similar at the initial priming of the CPB circuit (Table 2), significant differences were evident after the completion of PBUF (Tables 3a and 3b), which was anticipated. The first values on CPB continued to show the same trend of statistically significant differences for pH, sodium, glucose, lactate (Figure 1), and osmolality with the PBUF group having more physiologic values (Table 4). The last CPB values showed that most differences had equilibrated with the exception of glucose (Table 5, Figure 1). The pH of the SP cohort displayed a wider range of values throughout the measured time points compared to the PBUF cohort. Normality in a majority of measured blood parameters was evident by the end of CPB (Table 5).

**Table 2:** First prime laboratory values (each circuit initially primed with the same process).

	Standard Prime (*n* = 417)	PBUF (*n* = 704)	*P* value
pH at prime temperature of 30C (*n* = 417, 691)	7.37 [7.32, 7.43]	7.37 [7.32, 7.43]	0.84
Na+ (mmol/L) (*n* = 416, 691)	161 [159, 163]	161 [158, 163]	0.080
Glucose (mg/dL) (*n* = 416, 691)	338 [304, 369]	342 [307, 368]	0.40
K+ (mmol/L) (*n* = 417, 690)	5.3 [4.9, 5.8]	5.3 [4.8, 5.8]	0.93
Ca++ (mmol/L) (*n* = 416, 691)	0.88 [0.76, 1.02]	0.87 [0.72, 1.07]	0.80
Lactate (mmol/L) (*n* = 417, 691)	4.3 [3.5, 5.3]	4.5 [3.6, 5.4]	0.30
OSM (mOsm/kg H_2_O) (*n* = 394, 633)	341 [336, 346]	341 [335, 345]	0.75

Values shown are number (percent) or median [25th, 75th percentiles].

dL: deciliters; kg: kilograms; L: liters; mg: milligrams; mmol: millimoles; mOsm: milliosmoles; OSM: osmolality; PBUF: pre-bypass ultrafiltration.

**Table 3a:** Final prime laboratory values after PBUF for that group (no change for the SP group).

	Standard Prime (*n* = 417)	PBUF (704)	*P* value
pH at prime temperature of 30C (*n* = 417, 703)	7.37 [7.32, 7.43]	7.39 [7.34, 7.43]	0.004
Ca++ (mmol/L) (*n* = 416, 703)	0.88 [0.76, 1.02]	1.02 [0.91, 1.16]	<0.001
Na+ (mmol/L) (*n* = 416, 703)	161 [159, 163]	149 [147, 152]	<0.001
Glucose (mg/dL) (*n* = 416, 703)	338 [304, 369]	153 [135, 168]	<0.001
K+ (mmol/L) (*n* = 417, 703)	5.3 [4.9, 5.8]	4.1 [3.9, 4.4]	<0.001
Lactate (mmol/L) (*n* = 417, 703)	4.3 [3.5, 5.3]	2.1 [1.7, 2.6]	<0.001
OSM (mOsm/kg H_2_O) (*n* = 394, 649)	341 [336, 346]	307 [303, 313]	<0.001

Values shown are median [25th, 75th percentiles].

dL: deciliters; kg: kilograms; L: liters; mg: milligrams; mmol: millimoles; mOsm: milliosmoles; OSM: osmolality; PBUF: pre-bypass ultrafiltration.

**Table 3b:** Final prime laboratory values within more physiologic range.

	Standard Prime (*n* = 417)	PBUF (*n* = 704)	*P* value
Sodium in more physiologic range (140–150 mmol/L)	13 (3%)	437 (62%)	<0.001
Glucose in more physiologic range (<150 mg/dL)	8 (2%)	318 (45%)	<0.001
Potassium in more physiologic range (3.0–5.0 mmol/L)	150 (36%)	688 (98%)	<0.001
Lactate in more physiologic range (<3 mmol/L)	56 (13%)	612 (87%)	<0.001
OSM in more physiologic range (280–320 mOsm/kg)	23 (6%)	595 (92%)	<0.001

Values shown are number (percent).

**Table 4:** First CPB laboratory values.

	Standard Prime (*n* = 417)	PBUF (*n* = 704)	*P* value
pH on CPB^1^	7.33 [7.29, 7.37]	7.35 [7.31, 7.38]	<0.001
Na+ (mmol/L)	142 [141, 144]	141 [139, 143]	<0.001
Glucose (mg/dL) (*n* = 416, 704)	180 [158, 213]	154 [132, 178]	<0.001
K+ (mmol/L) (*n* = 417, 700)	3.5 [3.1, 4.0]	3.3 [3.0, 3.8]	0.001
Ca++ (mmol/L) (*n* = 416, 704)	1.18 [0.99, 1.34]	1.19 [1.04, 1.31]	0.71
Lactate (mmol/L) (*n* = 406, 699)	1.8 [1.4, 2.5]	1.5 [1.1, 2.1]	<0.001
OSM (mOsm/kg H_2_O) (*n* = 416, 704)	294 [290, 298]	290 [286, 295]	<0.001

Values shown are number (percent) or median [25th, 75th percentiles].

^1^pH on bypass was measured at 37 °C (alpha-stat) for all cases performed at >30 °C. pH on bypass was measured at the target temperature (pH-stat) for all cases ≤30 °C.

CPB: cardiopulmonary bypass; dL: deciliters; kg: kilograms; L: liters; mg: milligrams; mmol: millimoles; mOsm: milliosmoles; OSM: osmolality; PBUF: pre-bypass ultrafiltration.

**Table 5:** Last CPB laboratory values.

	Standard Prime (*n* = 417)	PBUF (*n* = 704)	*P* value
pH on CPB^1^	7.40 [7.37, 7.44]	7.40 [7.37, 7.44]	0.66
Na+ (mmol/L)	141 [139, 144]	140 [138, 143]	0.11
Glucose (mg/dL) (*n* = 416, 704)	181 [153, 212]	170 [141, 197]	<0.001
K+ (mmol/L) (*n* = 417, 700)	3.8 [3.5, 4.2]	3.9 [3.5, 4.2]	0.14
Ca++ (mmol/L) (*n* = 416, 704)	1.23 [1.09, 1.37]	1.21 [1.08, 1.33]	0.029
Lactate (mmol/L) (*n* = 406, 699)	2.1 [1.5, 3.0]	1.9 [1.5, 2.9]	0.12
OSM (mOsm/kg H_2_O) (*n* = 416, 704)	292 [287, 297]	290 [285, 296]	0.001

Values shown are number (percent) or median [25th, 75th percentiles].

^1^pH on bypass was measured at 37 °C (alpha-stat) for all cases performed at >30 °C. pH on bypass was measured at the target temperature (pH-stat) for all cases ≤30 °C.

CPB: cardiopulmonary bypass; dL: deciliters; kg: kilograms; L: liters; mg: milligrams; mmol: millimoles; mOsm: milliosmoles; OSM: osmolality; PBUF: pre-bypass ultrafiltration.

### Secondary outcomes

**Figure 2:**
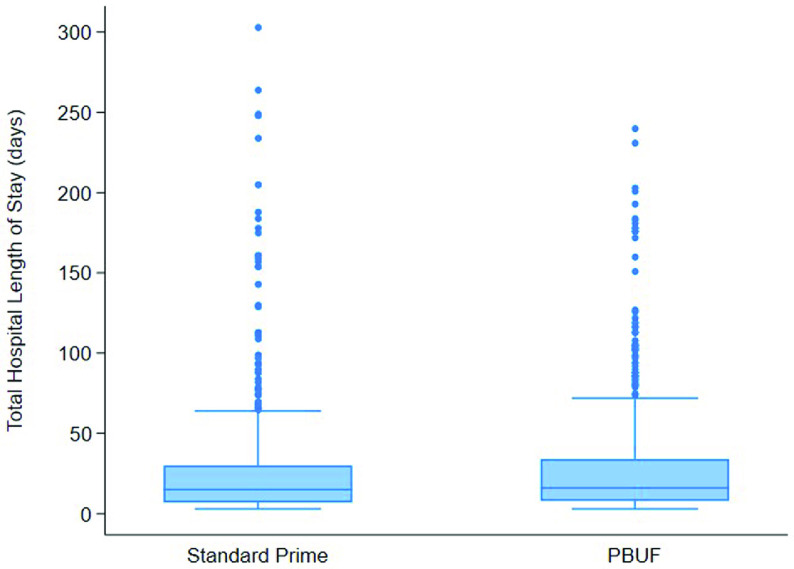
Total hospital length of stay. Distribution of total hospital lengths of stay is displayed as a box and whisker plot for both the standard prime and PBUF cohorts. The lower and upper borders of the box represent the 25th and 75th percentiles. The middle horizontal lines represent the median (Standard Prime = 15, PBUF = 16). The lower and upper whiskers represent the minimum and maximum values of non-outliers, while extra dots represent outliers. While Group PBUF exhibits a smaller range, there is no statistically significant difference between the two cohorts.

There were no statistically significant differences in clinical outcomes between the two cohorts for time to extubation, CICU LOS, unplanned reoperation, re-exploration for bleeding, mediastinitis, CNS complications, use of ECMO, pacemaker requirement, and mortality (Table 6). There were also no statistically significant differences in postoperative hospital LOS between the groups (Figure 2). Intraoperatively, the median dose of phenylephrine used while on bypass was 60 mcg for both cohorts (Table 7). The ultrafiltration total (modified ultrafiltration + conventional ultrafiltration) was no different between the two cohorts, and postoperative urine output was similar in the first 24 h (Table 7). There were no significant differences in the total dose/maximal infusion rate of inotropes perioperatively (Table 7).

**Table 6:** Clinical outcomes.

	Standard prime (*n* = 417)	PBUF (*n* = 704)	*P* value
Time to first extubation^1^ (hours)	50 [22, 124]	49 [21, 127]	0.96
CICU LOS (days)	5 [2, 11]	5 [2, 13]	0.64
Postoperative hospital LOS (days)	12 [6, 23]	12 [7, 27]	0.49
Unplanned reoperation (*n* = 416, 703)	41 (10%)	63 (9%)	0.67
Re-exploration for bleeding (*n* = 417, 702)	6 (1%)	12 (2%)	0.81
Mediastinitis (*n* = 417, 703)	2 (1%)	5 (1%)	1.0
CNS complication	11 (3%)	9 (1%)	0.11
ECMO	19 (5%)	22 (3%)	0.25
Pacemaker	14 (3%)	21 (3%)	0.73
Mortality	15 (4%)	26 (4%)	1.0

Values shown are number (percent) or median [25th, 75th percentiles].

^1^Begins at start of bypass.

CICU: cardiac intensive care unit; CNS: central nervous system; ECMO: extracorporeal membrane oxygenation; LOS: length of stay; PBUF: pre-bypass ultrafiltration.

**Table 7:** Additional operative and ICU characteristics.

	Standard Prime (*n* = 417)	PBUF (*n* = 704)	*P* value
Phenylephrine usage during bypass (mcg)	60 [4, 160]	60 [7, 156]	0.85
Ultrafiltration total (*n* = 405, 678)	500 [350, 750]	563 [350, 850]	0.17
ICU 24 hour output volume	414 [272, 567]	416 [307, 571]	0.26
*Preoperative Total Dose*			
Dopamine dose > 0 mg	10 (2%)	36 (5%)	0.029
Epinephrine dose > 0 mg	7 (2%)	28 (4%)	0.033
Milrinone dose > 0 mg	14 (3%)	21 (3%)	0.73
*Intraoperative Total Dose*			
Dopamine dose > 0 mg	327 (78%)	567 (81%)	0.40
Epinephrine dose > 0 mg	185 (44%)	349 (50%)	0.095
Milrinone dose > 0 mg	47 (11%)	80 (11%)	1.0
*Intraoperative Maximum Rate*			
Dopamine (mcg/kg/min) (*n* = 327, 567)	7 [5, 10]	7.5 [5, 10]	0.89
Epinephrine (mcg/kg/min) (*n* = 166, 297)	0.08 [0.05, 0.10]	0.08 [0.05, 0.10]	0.94
Milrinone (mcg/kg/min) (*n* = 45, 73)	0.50 [0.50, 0.75]	0.50 [0.50, 0.50]	0.55
*Postoperative Total Dose*			
Dopamine dose > 0 mg	131 (31%)	201 (29%)	0.31
Epinephrine dose > 0 mg	134 (32%)	263 (37%)	0.081
Milrinone dose > 0 mg	115 (28%)	192 (27%)	0.94
*Postoperative Maximum Rate*			
Dopamine (mcg/kg/min) (*n* = 147, 249)	5.0 [3.0, 5.0]	5.0 [3.0, 5.0]	0.60
Epinephrine (mcg/kg/min) (*n* = 142, 268)	0.05 [0.03, 0.07]	0.05 [0.02, 0.07]	0.93
Milrinone (mcg/kg/min) (*n* = 117, 191)	0.50 [0.50, 0.50]	0.50 [0.50, 0.50]	0.25

Values shown are number (percent) or median [25th, 75th percentiles].

hr: hours; ICU: intensive care unit; kg: kilograms; mcg: microgram; mg: milligrams; min: minutes; mu: milliunits; PBUF: pre-bypass ultrafiltration.

## Discussion

The process of blood priming CPB circuits is not standardized across institutions providing cardiopulmonary bypass for congenital heart surgery patients. Bank blood varies in degree of cell lysis and electrolyte composition based on duration and quality of storage. The use of PBUF provides more physiologic values for measured parameters and does so consistently. The method of PBUF used in this study intentionally did not normalize all values, especially the sodium and glucose, since some clinicians were concerned about an increased risk of edema after CPB given that the osmolality, primarily determined by sodium and glucose, decreased relative to the standard blood prime technique. Therefore, our technique of PBUF produced statistically significant improvements in measured values but not truly physiologic values (although glucose approached clinical significance), and this was by design. The last values on CPB were statistically similar, with the exception of glucose, and importantly there were no clinically significant differences. While the use of PBUF provided more physiologic prime values for glucose, sodium, potassium, and lactate that were statistically significant when compared to a standard prime, these advantages did not persist throughout the entire duration of the operation. The time spent on bypass was similar between groups and the median bypass time, which was over two hours, was likely sufficient for the patient’s compensatory mechanisms to correct most outlier values. Further, the similarity in final lab values may be in part explained by the fact that the patient may have been exposed to standard bank blood (when additional blood was clinically indicated) once on bypass since only the prime portion was treated with PBUF. Of note, the PBUF group preoperatively had a significantly greater proportion that required epinephrine and dopamine infusions. However, this was not adjusted for case complexity or preoperative risk factors. Further, intraoperative phenylephrine usage, intraoperative/postoperative inotropes, and patient outcome differences did not show statistical significance. Nonetheless, we believe the dataset provides us with confidence that making the CPB circuit prime more physiologic, and less hyperosmolar, does not increase patient risk. It could be argued that blood primes should be physiologic for patients requiring bypass unless there is compelling evidence otherwise. Our data set shows more values within the physiologic range throughout bypass and there were no adverse outcomes or events that occurred as a result of PBUF usage. We also found no evidence of a benefit with non-physiologic primes and are mindful that banked blood can have difficult to predict and dangerous levels of potassium, as reported by numerous authors [2–7]. We believe that since many electrolytes are known to be abnormal in prime blood, PBUF can consistently make these values more physiologic before bypass as well as throughout the cardiopulmonary bypass period.

Our dataset has led to additional practice changes. Our PBUF protocol now includes the entire unit of reconstituted blood for the procedure. Now, the entire unit of reconstituted whole blood is added to the circuit after a clear prime. PBUF is performed and then excess circuit volume, which has been PBUF treated, is returned to the original blood bag. Therefore, during CPB, the entire initial unit of blood that the patient is exposed to through the CPB circuit has been treated. See the Limitations section below. Additionally, we have modified the PBUF protocol to include more 0.45% saline and Plasma-Lyte, which results in more physiologic values. Besides what is measurable in a blood gas result, there are many other aspects addressed with our blood product preparations. Of note, our PRBC units are leukocyte-reduced, resulting in the patient being exposed to no more than 5,000,000 white blood cells (WBCs) per unit, per the American Association of Blood Banks (AABB) standards. This mitigates the incidence of febrile non-hemolytic transfusion reactions (FNHTRs) and transfusion-related acute lung injury (TRALI) which can be of significant benefit to morbidity and mortality scores [23]. Additionally, our RBC units are irradiated to 25 Gray units (Gy) in order to deactivate transfused lymphocytes and reduce the possibility of transfusion-associated graft versus host disease (TA-GVHD). Further, it is assumed that as a unit of blood ages, the activated neutrophils will continue to release their contents regardless of all preservative efforts including addition of Adsol and cold preservation [23]. In combination with leukocyte reduction, mitigating the deleterious impacts caused by the unavoidable exposure to activated WBCs should enhance recovery times by lessening endothelial dysfunction, complement activation, and the inflammatory response [23].

The use of a balanced electrolyte solution along with 0.45% saline during PBUF accomplishes two main objectives. One is to aid in the normalization of measured blood chemistry values, along with the requisite calcium gluconate and sodium bicarbonate added to the blood prime. Secondly, humoral factors such as cytokines, interleukins, and other cell signaling molecules are removed. It is worth noting that the 0.45% saline was not given to the patient during PBUF. Instead, it was administered to the blood prime to help account for the effects that the requisite sodium bicarbonate administration would have on prime sodium values.

Nagashima et al. applied the technique of PBUF to the blood prime pre-CPB for infants specifically undergoing an arterial switch operation [16]. In a preliminary study, they found that even when using outdated donated blood, which has far less physiologic values than anything that would normally be given to a patient, the electrolyte and acid-base balance can be dramatically improved in a time period as short as 30 minutes [16]. Specifically in this population, they saw that PBUF reduced cardiac impairment at early reperfusion periods and reduced pulmonary dysfunction. To note, our method of PBUF required approximately 15 minutes to perform.

### Limitations

It is important to note that our study was a retrospective review, which generally comes with limitations. One limitation of our study was that PBUF was performed only on the portion of the reconstituted blood unit that was included in the circuit prime. This equated to roughly half of the unit (approximately 200 mL of a 400–450 mL unit) being treated. This limitation may help explain why the primary outcome results were similar for the last values on bypass. Our updated protocol implemented after this study includes PBUF of the entire unit of reconstituted whole blood.

The updated protocol starts with a 3 IU/mL heparinized Plasma-Lyte prime for the 285–300 mL circuit to which the remainder (after a non-heparinized portion is given to the anesthesia team for PRN pre-bypass transfusion) of the 3 IU/mL heparinized reconstituted whole blood unit is then added. The 100% oxygen sweep gas is set to 0.4 LPM with 0.02 LPM CO_2_. The circuit volume is hemoconcentrated down to 250 mL and then 250 mL of additional Plasma-Lyte is added keeping the reservoir volume below the anti-foam coating level (500 mL in the Terumo CAPIOX FX 05 reservoir). The reservoir volume is then hemoconcentrated down to 150 mL and the hemoconcentrator effluent line is clamped. Sodium bicarbonate (17 [±1] mEq) is added for pH adjustment. Heparin (800 units) is added to help account for any losses in the effluent. Calcium gluconate (600 [±100] mg) is added for a target ionized calcium level of 0.8–1.2 mmol/L. Then, 100 mL of 0.45% saline is added. The additives are recirculated and then flow through the PBUF circuit is stopped. Approximately 175 mL of circuit volume is then sent back to the original blood unit bag for later transfusion while on bypass. A blood gas verifies that the prime values are within the target range. To note, this protocol results in a circuit prime hematocrit of approximately 20%. We have found that this commonly results in a dilutional hematocrit of approximately 30% once on bypass. Of course, the circuit prime volume can be further hemoconcentrated if a higher prime and/or dilutional hematocrit is desired.

Another limitation of our retrospective study is that while we found no statistically significant difference between groups in terms of overall ultrafiltration volumes, we could not control for perfusionist practice variation. We do know that on our large team, there is variability in how much conventional and zero balance ultrafiltration (ZBUF) one performs. The final lab values may have been similar between groups overall because of individual practice to perform ZBUF, and how much, particularly when additional blood was required during surgery.

This single-center study is also limited by the generalizability of our study findings to centers with a similar case mix and perfusion practice. This study shows that PBUF allows electrolyte values to correct to physiologic ranges more quickly, as opposed to waiting for the body to do this on its own. However, the statistically significant data that appear in final prime values does not automatically indicate that the change is clinically significant. Validation of these findings in a larger, preferably multicenter prospective cohort is warranted.

### Implications/next steps

The results suggest that PBUF is a useful and beneficial practice that can attenuate the perioperative stressors during infant cardiac surgery cases requiring CPB and a blood prime. Taking the load of correcting these electrolyte values off of the patient and putting it onto the perfusionist was a primary consideration when we started utilizing PBUF. It would be interesting to measure other elements of homeostasis during the perioperative period to determine where else PBUF may be beneficial. For example, avoidance of hypernatremia, starting with normalization of the sodium content of the prime components, may be particularly important in the protection of the neonatal brain. Rapid acute fluctuations in sodium levels and water movement may have detrimental sequelae such as cerebral edema, osmotic demyelination, and even intraventricular hemorrhage [24–26]. This is not well studied in neonatal and infant CPB cases but is certainly worthy of consideration when modifying priming procedures.

Furthermore, while the study indicates that the use of PBUF in a single surgical instance does not have any significant impact on immediate clinical outcomes, many single ventricle patients, for example, as well as congenital patients who require unplanned reoperations, must undergo CPB more than once. Looking at the cumulative effects of PBUF throughout these consecutive surgeries on patients and their long-term outcomes may demonstrate that PBUF is not only safe and effective but may also be beneficial in the long term. With one of the original concerns with PBUF being the possibility for negative neurologic implications as patients ages, a long-term review of patients who underwent PBUF would provide the clearest picture of its impact.

## Conclusion

The use of PBUF allows for more physiologic values for electrolytes, glucose, and lactate before the initiation of bypass without a negative impact on clinical outcomes or an increase in postoperative adverse events. PBUF creates predictable and consistent prime values when using bank blood products. We believe that PBUF is a safe and effective means to adjust blood prime values for neonates and infants undergoing surgery with CPB circuits requiring a blood prime.

## Glossary of abbreviations

AABB: American Association of Blood Banks
CCT: Cross-Clamp Time
CICU: Cardiac Intensive Care Unit
CNS: Central Nervous System
CPB: Cardiopulmonary Bypass
CUF: Conventional Ultrafiltration
ECMO: Extracorporeal Membrane Oxygenation
FNHTRs: Febrile Non-Hemolytic Transfusion Reactions
Gy: Gray Units
IU: International Units
LOS: Length of Stay
PBUF: Pre-Bypass Ultrafiltration
PRBC: Packed Red Blood Cells
RBC: Red Blood Cells
SP: Standard Prime
STAT: Society of Thoracic Surgeons-European Association for Cardio-Thoracic Surgery
TA-GVHD: Transfusion-Associated Graft Versus Host Disease
TPS: Technical Performance Score
TRALI: Transfusion-Related Acute Lung Injury
VAD: Ventricular Assist Device
WBCs: White Blood Cells
ZBUF: Zero Balance Ultrafiltration

## Data Availability

All available data are incorporated into the article.
